# A real-time and convex model for the estimation of muscle force from surface electromyographic signals in the upper and lower limbs

**DOI:** 10.3389/fphys.2023.1098225

**Published:** 2023-02-27

**Authors:** Mehdi Shirzadi, Hamid Reza Marateb, Mónica Rojas-Martínez, Marjan Mansourian, Alberto Botter, Fabio Vieira dos Anjos, Taian Martins Vieira, Miguel Angel Mañanas

**Affiliations:** ^1^ Automatic Control Department (ESAII), Biomedical Engineering Research Centre (CREB), Universitat Politècnica de Catalunya-Barcelona Tech (UPC), Barcelona, Spain; ^2^ Biomedical Engineering Department, Engineering Faculty, University of Isfahan, Isfahan, Iran; ^3^ Biomedical Research Networking Center in Bioengineering, Biomaterials, and Nanomedicine (CIBER-BBN), Madrid, Spain; ^4^ Laboratory for Engineering of the Neuromuscular System (LISiN), Department of Electronics and Telecommunication, Politecnico di Torino, Turin, Italy; ^5^ Postgraduate Program of Rehabilitation Sciences, Augusto Motta University (UNISUAM), Rio de Janeiro, Brazil

**Keywords:** electromyography, load sharing, convex optimization, artificial neural network, linear regression

## Abstract

Surface electromyography (sEMG) is a signal consisting of different motor unit action potential trains and records from the surface of the muscles. One of the applications of sEMG is the estimation of muscle force. We proposed a new real-time convex and interpretable model for solving the sEMG—force estimation. We validated it on the upper limb during isometric voluntary flexions-extensions at 30%, 50%, and 70% Maximum Voluntary Contraction in five subjects, and lower limbs during standing tasks in thirty-three volunteers, without a history of neuromuscular disorders. Moreover, the performance of the proposed method was statistically compared with that of the state-of-the-art (13 methods, including linear-in-the-parameter models, Artificial Neural Networks and Supported Vector Machines, and non-linear models). The envelope of the sEMG signals was estimated, and the representative envelope of each muscle was used in our analysis. The convex form of an exponential EMG-force model was derived, and each muscle’s coefficient was estimated using the Least Square method. The goodness-of-fit indices, the residual signal analysis (bias and Bland-Altman plot), and the running time analysis were provided. For the entire model, 30% of the data was used for estimation, while the remaining 20% and 50% were used for validation and testing, respectively. The average R-square (%) of the proposed method was 96.77 ± 1.67 [94.38, 98.06] for the test sets of the upper limb and 91.08 ± 6.84 [62.22, 96.62] for the lower-limb dataset (MEAN ± SD [min, max]). The proposed method was not significantly different from the recorded force signal (*p*-value = 0.610); that was not the case for the other tested models. The proposed method significantly outperformed the other methods (*adj. p*-value < 0.05). The average running time of each 250 ms signal of the training and testing of the proposed method was 25.7 ± 4.0 [22.3, 40.8] and 11.0 ± 2.9 [4.7, 17.8] in microseconds for the entire dataset. The proposed convex model is thus a promising method for estimating the force from the joints of the upper and lower limbs, with applications in load sharing, robotics, rehabilitation, and prosthesis control for the upper and lower limbs.

## 1 Introduction

Skeletal muscles generate forces to move different body parts or stabilize the skeleton (Basmajian and Luca, 1985). Handgrip force is one of the leading mechanical interactions between humans and the outside environment (Cao et al., 2017). For example, handgrip force has applications in opening doors, sports (Duc et al., 2008), military (Henning et al., 2011), and so on. Estimating the generated force in other body muscles like forearm muscles, biceps, triceps, and lower limbs is essential. The amputation of the lower limb (toe/foot) increased in the last few years (Spoden et al., 2019). Also, diseases like stroke, spinal cord injury, and other disabling disorders can cause disabilities. With the increment of disabilities, a prosthesis that can compensate for a lost limb is necessary. Surface electromyography (sEMG) could control advanced prosthesis in amputees (myoelectric-controlled prosthesis).

The sEMG is a signal consisting of different motor unit action potential trains and is recorded from the skin. Because of the possibility of misalignment of the electrode pair in conventional sEMG recordings and muscle fiber direction (Staudenmann et al., 2006), in addition to the fact that a pair of electrodes may not fully and accurately represent the muscle activity and be vulnerable to the innervation zone (IZ) effect, using High-Density sEMG (HD-sEMG) is beneficial. In HD-sEMG, we can record from hundreds of skin points, covering the target muscle’s whole or most volume. HD-sEMG has applications in gait and movement analysis, myoelectric control (Wang et al., 2017), biofeedback (Huang et al., 2013), fatigue evaluation (Cifrek et al., 2009), gesture recognition (Du et al., 2010), obstetrics, occupational medicine, aging, rehabilitation, gaming, ergonomics, and force estimation (Merletti and Muceli, 2019). One of the applications of HD-sEMG and sEMG is the estimation of muscle force (Staudenmann et al., 2005). The generation of force is always related to the electrical activity of the HD-sEMG with limitations to superficial muscles or motor units, and it depends on the recruitment of motor units and the firing rate of active motor units (Erim et al., 1996). Other factors that affect the sEMG are muscle length and the IZ location. Estimation of muscle force has various applications in biomechanics and kinesiology (Christophy et al., 2012), exoskeleton control (Lenzi et al., 2012; Li et al., 2014), prosthesis control (Castellini and Van Der Smagt, 2009), grasping force (Gurari and Okamura, 2007; Farina and Holobar, 2015), and military (Henning et al., 2011).

Many studies attempted to explain the EMG-force relationship and estimate force in different muscles based on the sEMG signal. Different studies were performed on biceps and triceps muscles (Buchanan et al., 1993; Park and Meek, 1995; Luh et al., 1999; Clancy et al., 2001; Potvin and Brown, 2004; Staudenmann et al., 2005; Mobasser et al., 2007; Staudenmann et al., 2007; Nielsen et al., 2010; Botter et al., 2011; Hashemi et al., 2012; Allouch et al., 2013; Hashemi et al., 2013, 2014; Li et al., 2014; Na and Kim, 2016; Huang et al., 2017; Xu et al., 2018), forearm muscles (Youn and Kim, 2010; Liu et al., 2014), hand griping (Cao et al., 2017), and lower limbs (Hayashibe et al., 2009; Amarantini et al., 2010; Menegaldo and Oliveira, 2012; Hayashibe and Guiraud, 2013; Cao et al., 2015; Rane et al., 2019). Different modeling of the problem was considered in these studies, and various solutions were presented. Ma et al. (Ma et al., 2020) predicted grasping force based on the sEMG with the gene expression programming (GEP) and also compared the result of the GEP algorithm with the Back Propagation (BP) neural network algorithm. The GEP algorithm achieved a better overall result than their study’s BP neural network algorithm. They predicted grasping force in 20%, 40%, 60%, and 80% MVC and reported root mean square error (RMSE) and correlation coefficient (CC) in their work. In the 60% MVC, they achieved 7.5% RMSE and 95% CC. Chen et al. (2020) used three degrees of freedom (DoF) of finger movement to predict force from HD-sEMG based on only one DoF (doing all of the finger movements sequentially in one movement) in the training part. They used Convolutional Neural Network (CNN) and Recurrent Neural Network (RNN) methods, with minimal numbers of trials (using 1-DoF trials only) to train the model and then assessed on multi-DoF trials. The muscle crosstalk was reduced using HD-sEMG, and the entire recording channels were used. The authors showed that multi-DoF control for individual fingers is possible with minimal training. Rane et al. (2019) predicted force from the lower limb muscles with deep learning and reported Pearson’s correlation coefficient (*r*) and RMSE for fitness criteria. The best value of *r* was 0.91, and RMSE was 126 (N) for the hamstring muscles in all test trials. Xu et al. (2018) approached the force estimation problem in the biceps brachii muscle with the CNN and long short-term memory (LSTM) and their combination. The best model for the 50% MVC had a %RMSE of 5.69.

Various performance indices were proposed in the literature for the EMG-force problem. For example, Huang et al., (2017) used a non-negative matrix factorization algorithm and a polynomial model to estimate muscle force from the biceps brachii muscle. They report % root mean square difference (RMSD), variability accounted for (VAF), and the correlation coefficient in different figures for all subjects in their studies. Cao et al. (2017) used extreme machine learning (EML) algorithm to predict handgrip force using the EMG signal of forearm muscles. They compared their algorithm with the support vector machine (SVM) and multiple non-linear regression (MNLR) and reported a comparable result in time and accuracy with these algorithms. For the evaluation of the results, they used RMSE and CC. Na et al. (Na and Kim, 2016) estimated elbow flexion force using a muscle-twitch model with sEMG in the fatigue condition and then compared their results with the mean absolute value (MAV) method. To evaluate the results, they used $R^{2}$ and %RMSE criteria. Hashemi et al. (2014) used an angle-based EMG calibration method and parallel cascade identification (PCI) modeling to estimate muscle force in elbow flexion and extension in different angles. They evaluated their results with %RMSE criteria. The summary of the EMG-force estimation algorithms and performance indices is presented in Supplementary Table S1.

The EMG-force problem is a regression problem in which regression diagnostics (e.g., residual signal analysis (O'Connor et al., 2011; Giavarina, 2015) are essential in addition to the goodness-of-fit indices. Moreover, a proper statistical method is required for rigorous comparison of the proposed and state-of-the-art methods. Otherwise, bias occurs (Davidson and MacKinnon, 1981).

In such studies, specific muscles were used, and the generalization ability of such algorithms was not discussed. Thus, there is a need for a new method that can be robust enough to be used in different muscles and fast enough for real-world applications.

Many approaches have been investigated in the literature for solving the EMG-force relationship problems until now, including the Hill model (Hill, 1938; Hayashibe et al., 2009; Hayashibe and Guiraud, 2013), fast orthogonal search (Mobasser et al., 2007), polynomial fitting model (Clancy and Hogan, 1997), parallel cascade identification (Hashemi et al., 2012), different neural network architectures (Choi et al., 2010; Mobasser and Hashtrudi-Zaad, 2012; Su et al., 2021), non-negative matrix factorization (NMF) (Huang et al., 2017).

Hill-type models have various problems. There is an error in estimating force from EMG when there are different firing frequencies, activation levels, and contraction speeds (Perreault et al., 2003; Hayashibe and Guiraud, 2013). Hill’s model is mainly based on macroscopic modeling and does not relate to microscopic physiology. Besides the large error in different firing frequencies, the error in the low motor unit firing rates is very high (Perreault et al., 2003). Moreover, Hill’s model is not usually practical when high, and low force contraction is simultaneously considered because each of them needs to set the cut-off frequency of the low pass filter differently (Hermens et al., 1999; Perreault et al., 2003; Hayashibe and Guiraud, 2013). On the other hand, polynomial fitting models tend to overfit when the number of involved muscles increases (such as the lower limb dataset), and their prediction error significantly increases (Clancy and Hogan, 1997). Also, in the NMF methods, the identification of features depends strongly on the exact dataset and on converging to a set of highly sparse factors (Burkholder and van Antwerp, 2013).

In this paper, we propose a new real-time convex algorithm to estimate muscle force for the lower and upper limbs, and thorough regression diagnostics and rigorous statistical comparison with the state-of-the-art are provided.

## 2 Materials and equipment

In this study, we used two of our previously recorded datasets. The first set is related to the lower limb (dos Anjos et al., 2017), while the second is HD-sEMG and force in the upper limb (Botter et al., 2011). The full description of the experimental protocol of both datasets is available in the original papers, and here we describe them briefly.

### 2.1 Participants

In the lower limb dataset, we recorded data from nineteen young volunteers (14 male and 5 females, with age 26.0 ± 3.0 (MEAN ± SD) (years)) and fourteen aged volunteers (12 male and 2 female, with age70.0 ± 6.0 (years)). The physical activity of the participants was assessed according to the international physical activity questionnaire (IPAQ) (Booth, 2000), and they were then ranked with minimally active (low) or active (moderate or high) scores (Sember et al., 2020). Based on volunteer reports, none of them did have any balance impairment, neurological disorders, muscular injuries, or the intake of medications for body balance.

In the upper limb dataset, we recorded data from five healthy male volunteers with an average age of 21.3±2.8 (years), weight of 71.0 ± 3.4 (kg), and height of 174.3 ± 2.6 (cm).

In both datasets, all subjects gave informed consent to the experimental procedure. The procedures were confirmed with the Declaration of Helsinki and were approved by Politecnico di Torino Research Ethics Committee.

### 2.2 Experimental setup

**FIGURE 1:**
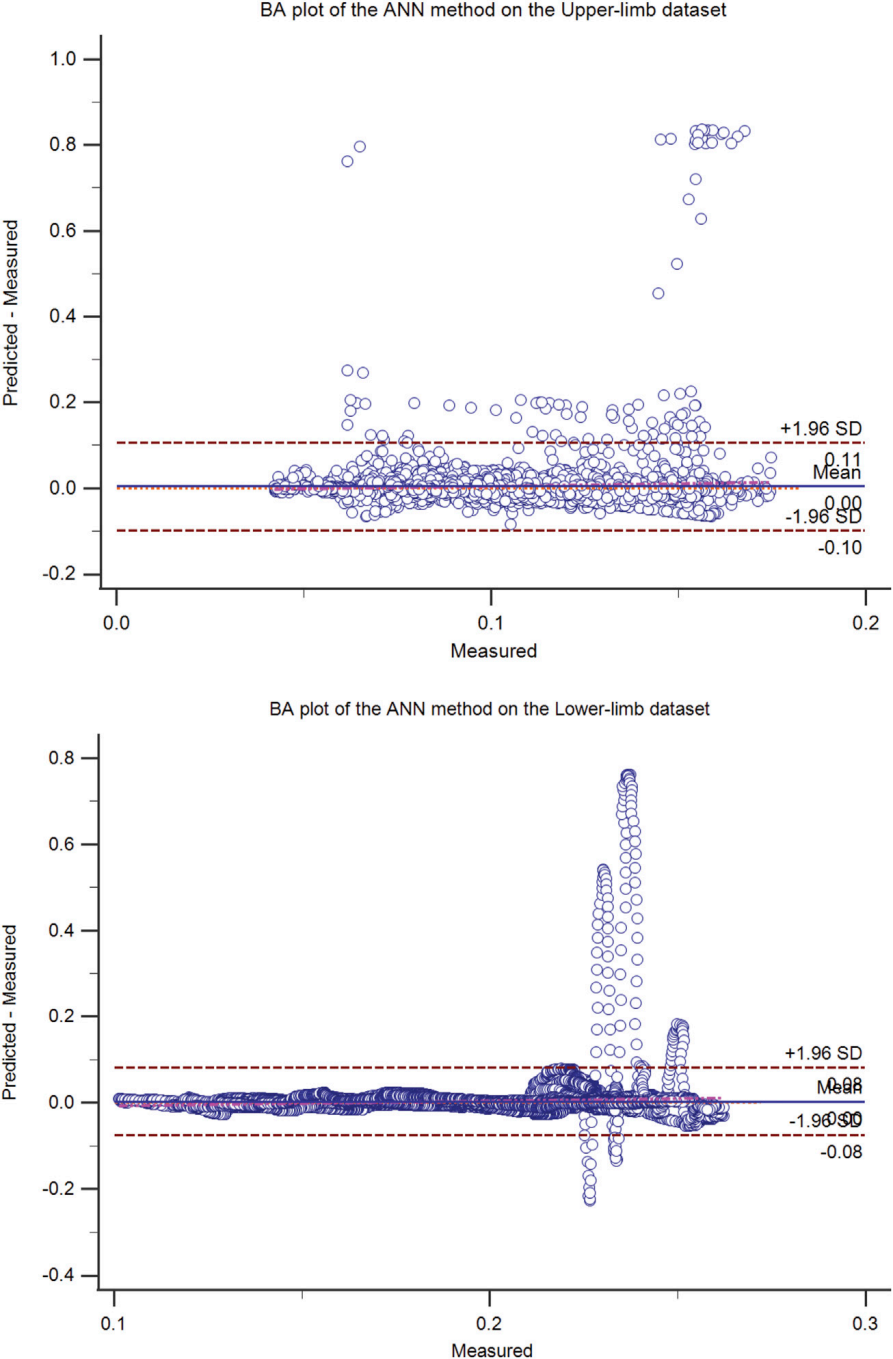
The Bland-Altman (BA) plot of the ANN method on the upper-limb (top) and the lower-limb (bottom) test dataset (50% hold-out validation). The BA plot shows the scatter plot of the residual signal (predicted force minus measured force signal) (y-axis) and the measured force signal (x-axis), which identifies the homogeneity of the residual signal at different measured signal levels. In addition to the mean residual signal (i.e., bias), the upper and lower boundaries (mean (residual signal) ± SD (residual signal)) are also shown.

In the lower limb dataset, sEMG signals were recorded in signal differential mode. All signals were amplified by a between-individuals variable factor, ranging from 5,000 to 10,000, to maximize the analog gain without saturation. The force signals were recorded by a piezoelectric force plate (9286AA Kistler, Zurich, Switzerland). Both sEMG and force signals were sampled synchronously at 2048 Hz with a 12-bit analog-to-digital converter (EMG-USB, OTBioelettronica, and LISiN, Politecnico di Torino, Turin, Italy). We used linear array electrodes to record the calf muscles’ activity: sEMG from tibialis anterior and medial and lateral gastrocnemius muscles were detected with three arrays of 16 electrodes, each with 10 mm inter-electrode distance (IED), whereas two arrays of four electrodes each with 10 mm IED were used to sample EMGs medially and laterally from soleus (cf Figure 1) in (dos Anjos et al., 2017). For the gastrocnemius muscle, the most proximal electrode was located 2 cm distal to the popliteal fossa, and the arrays were aligned parallel to the longitudinal axis of each gastrocnemius head. For the tibialis anterior muscle, the array was aligned 1 cm laterally and parallel to the tibial crest, with the most proximal electrode located 2 cm distal to the fibula’s head. The soleus muscle’s lower border of the medial and lateral arrays was positioned 3 cm distal to the medial gastrocnemius myotendinous junction (dos Anjos et al., 2017).

In the upper limb dataset, sEMG signals were recorded from the Biceps Brachii (BB), Brachioradialis (BR), Triceps Brachii lateral (TBL), and medial head (TBM) during isometric voluntary flexions-extensions with the angle of the elbow at 90 degrees. For recording from BB, we used an HD-sEMG array of 64 circular electrodes disposed into five columns and 13 rows with 8 mm IED (one missing corner electrode). For BR, TBL, and TBM recording, we used three linear arrays of 8 electrodes with an IED of 5 mm. The main IZ was located for each muscle before placing the electrode array. Depending on the subject’s anatomical features, we placed the adhesive arrays either proximally or distally from the main IZ location. We used paste to clean the skin before the recording (Meditec-every, Parma, Italy). We recorded data in monopolar mode with the same amplifier used in the lower limb experiments, allowing for the synchronous recording of HD-sEMG and force signals after amplifying the latter by a factor of 100 (Force Amplifier MISO-II, LISiN, Politecnico di Torino, Italy). The force signal was shown to the subjects as real-time feedback (Botter et al., 2011).

### 2.3 Experimental Protocol

In the lower limb experiments, participants were asked to stand upright on a force plate with eyes open and arms alongside the body. Ground reaction forces were measured in two conditions while subjects: i) kept the position of their center of pressure at 65% of the distance between the tip of the calcaneus bone and the tip of the third metatarsal head and; ii) stood at ease. Both trials lasted 60 s, and center-of-pressure visual feedback was provided in the first trial. This task was selected to ensure a somewhat high degree of calf muscle active loading while not threatening stability, particularly for aged individuals. Five-minute intervals were applied between trials, and their order was randomized.

In the upper limb dataset, each of the volunteers was asked to do three maximum voluntary isometric flexions and extension contractions for 5 s before starting the experiment, and the highest was chosen as the Maximum Voluntary Contraction (MVC). After a few pieces of training for each subject, they were asked to perform a series of flexion-extension force ramps for 25 s. Each series consisted of four isometric ramps from different percentages (30%, 50%, and 70%) extension MVC to flexion MVC and back. The single differential signals were used to reduce muscle crosstalk (Botter et al., 2011; Jafari et al., 2014).

## 3 Methods

### 3.1 Data Processing

The lower limb dataset was recorded in single differential mode, and the signals were digitally bandpass filtered with a fourth-order Butterworth filter in the 15–350 frequency band. Given that we were interested in estimating the temporal force profile, regardless of its absolute value, the center of pressure position in the sagittal plane was taken as the ankle force signal (Morasso and Schieppati, 1999). In the upper limb dataset, the monopolar HD-sEMG signals were digitally bandpass filtered (20–450 Hz with a fourth-order Butterworth filter), and the force signal was low pass filtered (cut off at 1 Hz with a fourth-order non-casual Butterworth filter).

### 3.2 Method description

This section briefly introduces the least square (LS) problem and its solution based on calculus.

Suppose that we have linear equations Ax=b , where matrix A has n rows (i.e., equations) and m columns (i.e., unknowns), *x* is the unknown vector with *m* rows, and b is an n−vector . These equations have a solution if b is a linear combination of columns of A .

For most cases, we need to find an x for r=Ax−b , that minimizes the residuals. We choose x that can minimize the norm of the residual, $\left\|Ax-b\right\|$ . Minimizing the norm of the residual is similar to minimizing its square. The problem of finding m−vector $\hat{x}$ that minimize ${\left\|Ax-b\right\|}^{2}$ is called a least squares problem.

$$\hat{x}={\mathrm{argmin}}_{x}{\left\|Ax-b\right\|}^{2}$$ (1)

This residual is an affine function. Affine functions are considered linear functions, and they are convex. To solve this problem, we must calculate the gradient.

$$\frac{\partial f}{\partial x_{i}}\left(\hat{x}\right)=0,i=1,\ldots ,m$$ (2)

Or we can consider it in vector notation

$$\nabla f\left(\hat{x}\right)=0.$$ (3)

The gradient in the matrix form is described below

$$\nabla f\left(x\right)=2A^{T}\left(Ax-b\right)=0$$ (4)

The solution can be presented as the following:

$$A^{T}A\hat{x}=A^{T}b$$ (5)

This equation is called a normal equation, and with the assumption that columns of A are linearly independent, the solution is calculated as below:

$$\hat{x}={\left(A^{T}A\right)}^{-1}A^{T}b$$ (6)

If the matrix A is not full rank, the normal equation must be solved with Moore-Penrose pseudo-inverse with the help of the singular value decomposition (SVD) (Strang et al., 1993). When the SVD is calculated, the reciprocal of the non-zero items of the middle matrix is calculated while keeping the zero items, and multiplying SVD matrices from right to left is then used as the pseudo-inverse matrix.

#### 3.3 The proposed mathematical model

This section proposes a new method for estimating the muscle force from sEMG signals. The following relationship between muscle force and activity is used in our study:

$$f\left(t\right)=w_{0}+\overset{M}{\underset{i=1}{\sum }}w_{i}\times \ln\left({sEMG}_{i}\left(t\right)\right)$$ (7)

t∈R,w∈R,M∈N

where, $f\left(t\right)$ is the muscle force, M is the number of muscles, $sEMG_{i}\left(t\right)$ is the envelope of the i muscle, and $w_{i}$ is the weight of the $i^{th}$ muscle. The sEMG envelope was estimated by low-pass (LP) filtering of the full-wave rectified sEMG signals. A second-order zero-lag Butterworth LP filter with a cut-off frequency of 2.0 Hz was used in our study, based on the SENIAM recommendations is 2 Hz for slow motions (Hermens et al., 1999). The median envelope of the sEMG signals was then used as a representative envelope of the analyzed muscle.

As we have discrete-time samples of the EMG and force signals, the following notation is produced for *N* samples:

$$\left[\begin{array}{c} \begin{array}{c} f\left[1\right]\\ .\\ . \end{array}\\ \begin{array}{c} .\\ .\\ f\left[N\right] \end{array} \end{array}\right]=\left[\begin{array}{c} \begin{array}{cc} \begin{array}{cc} \begin{array}{c} 1\\ .\\ . \end{array} & \begin{array}{cc} \begin{array}{c} \ln\left({sEMG}_{1}\left[1\right]\right)\\ .\\ . \end{array} & \begin{array}{c} .\\ .\\ . \end{array} \end{array} \end{array} & \begin{array}{cc} \begin{array}{cc} \begin{array}{c} .\\ .\\ . \end{array} & \begin{array}{c} .\\ .\\ . \end{array} \end{array} & \begin{array}{c} \ln\left({sEMG}_{M}\left[1\right]\right)\\ .\\ . \end{array} \end{array} \end{array}\\ \begin{array}{cc} \begin{array}{cc} \begin{array}{c} .\\ .\\ 1 \end{array} & \begin{array}{cc} \begin{array}{c} .\\ .\\ \ln\left({sEMG}_{1}\left[N\right]\right) \end{array} & \begin{array}{c} .\\ .\\ . \end{array} \end{array} \end{array} & \begin{array}{cc} \begin{array}{cc} \begin{array}{c} .\\ .\\ . \end{array} & \begin{array}{c} .\\ .\\ . \end{array} \end{array} & \begin{array}{c} .\\ .\\ \ln\left(sEMG_{M}\left[N\right]\right) \end{array} \end{array} \end{array} \end{array}\right]\left[\begin{array}{c} \begin{array}{c} w_{0}\\ w_{1}\\ . \end{array}\\ \begin{array}{c} .\\ .\\ w_{M} \end{array} \end{array}\right]$$ (8)

We can rewrite the formula 8 in the form of LS ( Ax=b) , whose solution is provided by (6).

#### 3.4 State-of-the-art

Various models were proposed in the literature to estimate the force signal. We provided such mathematical models in Eqs 9–17 (Nurhanim et al., 2013; Nurhanim et al., 2014), shown as models 1-9.

$$Model\;1:TR=\overset{M}{\underset{i=1}{\sum }}a_{i}\times sEMG_{i}+b_{i}\times \sqrt{sEMG_{i}}$$ (9)

$$Model\;2:TR=\overset{M}{\underset{i=1}{\sum }}a_{i}{\times sEMG}_{i}^{b_{i}}$$ (10)

$$Model\;3:TR=\overset{M}{\underset{i=1}{\sum }}a_{i}\times e^{\frac{b_{i}}{sEMG_{i}}}$$ (11)

$$Model\;4:TR=\sum_{i=1}^{M}a_{i}{\times sEMG}_{i}^{b_{i}}+c_{i}{\times sEMG}_{i}^{d_{i}}$$ (12)

$$Model\;5:TR=\sum_{i=1}^{M}a_{i}{\times sEMG}_{i}^{4}+b_{i}{\times sEMG}_{i}^{3}+c_{i}\times {sEMG}_{i}^{2}+d_{i}\times {sEMG}_{i}^{1}+e_{i}$$ (13)

$$Model\;6:TR=\overset{M}{\underset{i=1}{\sum }}a_{i}+b_{i}\times \sqrt{sEMG_{i}}$$ (14)

$$Model\;7:TR=\overset{M}{\underset{i=1}{\sum }}{sEMG}_{i}^{a_{i}}+e^{\left(b_{i}-c_{i}sEMG_{i}\right)}$$ (15)

$$Model\;8:TR=\overset{M}{\underset{i=1}{\sum }}a_{i}+b_{i}\times \cos\left(sEMG_{i}\right)+c_{i}\times \sin\left(sEMG_{i}\right)$$ (16)

$$Model\;9:TR=\overset{M}{\underset{i=1}{\sum }}a_{i}+b_{i}\times \sin\left(sEMG_{i}\right)$$ (17)

where, *TR* is the estimated force, sEMG_i_ is the representative envelope of the sEMG signal of the *i*th muscle, *M* is the number of muscles, and *a*_*i*_ *, b*_*i*_ *, c*_*i*_ *, d*_*i*_ *,* and *e*_*i*_ are the unknown coefficients of the *i*th muscle. Models 1, 5, 6, 8, and 9 are Linear-in-the-parameters models whose solutions are provided by the LS method. However, models 2, 3, 4, and 7 are not convex, and their parameters were estimated using Particle Swarm Optimization (PSO), a meta-heuristics population-based stochastic optimization algorithm (Botter et al., 2011).

Also, other methods proposed in the literature were implemented for comparison, including Ordinary Least Squares (OLS) (Chatterjee and Hadi, 1986; Ziai and Menon, 2011), Regularized Least Squares (RLS) (Kim et al., 2007; Xue et al., 2009; Ziai and Menon, 2011), Support Vector Machine (SVM)(Castellini and Van Der Smagt, 2009), and Artificial Neural Network (ANN) (Setiono and Hui, 1995; Lin and Buchanan, 2002; Castellini and Van Der Smagt, 2009; Ziai and Menon, 2011).

The OLS method is based on the classical LS, which assumes a linear combination of the muscle activity maps. The support vector regression (SVR) was used as the extension of SVM to regression problems. The linear SVM was used in our study, and the penalty parameter was tuned using cross-validation on the estimation set (Smola and Schölkopf, 2004; Chen et al., 2006). A feedforward ANN with ten hidden layers and mean squared error (MSE) loss function, Marquardt-Levenberg modification to the Gauss-Newton algorithm (Hagan and Menhaj, 1994) with the initial learning rate of 0.001 (with adaptive decrease and increase approaching the Gauss-Newton to the steepest descent algorithm in borderlines (Martin et al., 2000) were used in our study.

Note that the proposed model, OLS, and models 1,5,6,8, and 9 are linear-in-the-parameters (LIP) models since the output is a linear combination of the model parameters, and any non-linear input function can be used as their weight. Such models can be solved by the LS, resulting in a global minimum (Pintelon and Schoukens, 2012). Moreover, LIP models (a.k.a. Affine functions) are convex (Beck, 2014).

#### 3.5 Evaluation criteria

In this study, we used Pearson Correlation Coefficients (*r*), coefficient of determination ( $R^{2}$ ) and adjusted R-squared (*adj. R* ^2^) for the goodness-of-fit between the original and the estimated force signal from the sEMG signals on the test set. (Pearson’s) Correlation is a criterion to show similarity:

$$r=\frac{\overset{N}{\underset{i=1}{\sum }}\left(y_{i}-\bar{y}\right)\times \left({\hat{y}}_{i}-{\hat{y}}_{m}\right)}{\sqrt{\left({\overset{N}{\underset{i=1}{\sum }}\left(y_{i}-\bar{y}\right)}^{2}\right)\times \left(\overset{N}{\underset{i=1}{\sum }}{\left({\hat{y}}_{i}-{\hat{y}}_{m}\right)}^{2}\right)}}$$ (18)

where, $y_{i}$ is the measured and ${\hat{y}}_{i}$ is estimated force signals, $\bar{y}$ and ${\hat{y}}_{m}$ are the average values of the measured and estimated force signals, respectively.

The coefficient of determination ( $R^{2}$ ) is defined as the following:

$$R^{2}=1-\frac{SS_{E}}{SS_{T}}$$ (19)

where, $SS_{E}$ is error sum of squares:

$$SS_{E}=\overset{N}{\underset{i=1}{\sum }}e_{i}^{2}=\overset{N}{\underset{i=1}{\sum }}{\left(y_{i}-{\hat{y}}_{i}\right)}^{2}$$ (20)

And, $SS_{T}$ is the total sum of squares:

$$SS_{T}=\sum_{i=1}^{N}\left(y_{i}-\bar{y}\right)$$ (21)

Moreover, as the number of parameters of the analyzed models are different, the adjusted R-squared (adj. *R* ^2^) was also reported, defined as the following:

$$adj.\;R^{2}=1-\left(1-R^{2}\right)\times \frac{N-1}{N-k-1}$$ (22)

where, k is the number of parameters of the model to estimate.

The Bland and Altman plot was also used to describe the agreement between the estimated and measured force signals. The result is a scatter plot, where Y-axis shows the difference between two measurements and X-axis shows the measured signal samples (Bland and Altman, 1999; Giavarina, 2015).

#### 3.6 Data allocation strategy

For models 2, 3, 4, 7, SVM, RLS, and ANN, 30% of the data was used for estimation, while the remaining 20% and 50% were used for validation and testing, respectively. These methods required cross-validation on the training set (e.g., SVM, and RLS) to tune free parameters or run PSO several times to select the best fit. For the proposed algorithm and models 1, 5, 6, 8, 9, and OLS, 50% of the data was used for estimation, and the remaining 50% was used for testing. It thus provided the hold-out validation (50%) for the entire model. The analysis and comparison of the results of different methods were performed on the test set.

The parameters of the PSO algorithm were tuned as the following: The maximum iteration number was set to $\left(200\times number\;of\;model\;weights\right)$ . Minimum adaptive neighborhood size, self-adjustment weight, social adjustment weight, and swarm size were set to 0.25, 1.49, 1.49, and $\min\left(100,10\times number\;of\;model\;weights\right)$ , respectively (Engelbrecht, 2007). The PSO algorithm was run ten times, and the models with the best validation results were used. In this procedure, the estimation set was used to estimate the EMG-force parameters, and the RMSE of the predicted force compared with the measured force signal on the validation set was calculated. Since PSO is a stochastic optimization method, it could have different results at different runs. The model with the lowest RMSE was then selected among the ten runs. The interior-point method proposed by was used to solve regularized least squares (RLS) Kim et al. (2007). The lambda parameter was set to 0.01 based on trial and error on the estimation and validation sets. The analysis was performed on an Intel Core i7-8750H with 2.21 GHz CPU with 16 GB of RAM. The results of the running time analysis are provided in MEAN ± SD [min., max.].

#### 3.7 Statistical analysis

Results are reported as mean ± standard deviation. The normality of the data was tested using the Shapiro-Wilk test. Due to the normality of the data, different models were compared using repeated-measures analysis of variance (rm-ANOVA). The Bonferroni correction was used for a pairwise comparison between the proposed method and the state-of-the-art. The independent-samples *t*-test was used to compare the Mean Absolute Error (MAE) of the proposed method in the young and elderly groups of the lower-limb dataset. The paired-sample *t*-test was used to identify whether the proposed method has a significant bias (Mansourian et al., 2020). The association between two normally-distributed variables was assessed using Pearson’s correlation coefficient (*r*).

The Mann-Whitney *U* test was used to compare differences between the eleven weights of the lower-limb model in two (minimally active and active) independent groups. When the Mann-Whitney *U* test was significant (discriminative features), the receiver operating characteristic (ROC) curve was provided. The Area under the ROC Curve (AUC) was also provided for discriminative features. The best ROC cut-off was estimated for each discriminative feature using Youden index J. The AUC of such features was the tested using the method proposed by DeLong et al. (1988).

The level of statistical significance was set to *p*-value = 0.05. All data processing was performed offline using MATLAB version 9.10 (The MathWorks Inc., Natick, MA, USA). All statistical analysis and calculations were performed using IBM SPSS Statistics version 27 (IBM Corp).

## 4 Results

The performance of the different system identification methods is presented for the upper-limb (Table 1) and lower-limb (Table 2) datasets. Analyzed methods had statistically significantly different results (F (4,567728) = 969.856; *p*-value < 0.001). The proposed algorithm significantly outperformed the other methods (*adj. p*-value < 0.05). The performance of the proposed method was significantly higher in the young groups compared with the older groups of the lower-limb dataset (*p*-value < 0.001). The proposed method did not have a significant bias in the entire dataset (*p*-value = 0.610).

**TABLE 1:** The results of different models on the test set of the upper limb dataset in Mean ± SD [minimum, maximum] (50% hold-out validation).

Model	Correlation (×100%)	$R^{2}$ (×100%)	Adj. *R* ^2^ (×100%)
1	97.70 ± 1.67 [94.95, 99.42]	95.54 ± 3.25 [90.16, 98.84]	95.59 ± 3.25 [90.17, 98.85]
2	83.66 ± 15.32 [60.04, 96.19]	71.86 ± 24.14 [36.05, 92.52]	71.87 ± 24.14 [36.05, 92.53]
3	1.22 ± .07 [1.15, 1.33]	0.01 ± 0.00001 [0.01, 0.017]	0.01 ± 0.00001 [0.01, 0.017]
4	90.27 ± 5.76 [83.23, 97.38]	81.75 ± 10.42 [69.26, 93.97]	81.77 ± 10.42 [69.28, 94.00]
5	31.36 ± 11.67 [14.40, 43.76]	15.50 ± 1.79 [0.09, 26.09]	15.51 ± 1.79 [0.09, 26.10]
6	99.14 ± 0.41 [98.43, 99.38]	98.29 ± 0.80 [96.88, 98.76]	98.30 ± 0.80 [96.89, 98.77]
7	74.87 ± 15.68 [56.76, 93.09]	50.03 ± 23.34 [32.22, 86.66]	50.04 ± 23.34 [32.22, 86.67]
8	42.19 ± 41.99 [5.30, 86.15]	31.91 ± 32.04 [0, 74.22]	31.92 ± 32.04 [0, 74.23]
9	33.27 ± 37.81 [7.64, 76.08]	22.51 ± 23.87 [0.32, 57.88]	22.51 ± 23.87 [0.32, 57.89]
OLS	94.14 ± 3.01 [89.12, 96.72]	88.70 ± 5.58 [79.40, 93.56]	88.70 ± 5.58 [79.40, 93.56]
RLS	94.14 ± 3.01 [89.11, 96.73]	88.70 ± 5.58 [79.40, 93.56]	88.70 ± 5.58 [79.40, 93.56]
ANN	87.21 ± 20.01 [51.56, 97.49]	79.26 ± 29.63 [26.59, 95.04]	79.24 ± 29.56 [26.51, 95.04]
SVM	95.49 ± 2.69 [91.38, 98.16]	91.24 ± 5.11 [83.50, 96.36]	91.24 ± 5.10 [83.50, 96.36]
The proposed algorithm	98.36 ± 0.85 [97.15, 99.03]	96.77 ± 1.67 [94.38, 98.06]	96.77 ± 1.67 [94.38, 98.06]

The Correlation Coefficient and Goodness-of-fit measures (*R* ^2^ and *adj. R* ^2^) were calculated between the reconstructed and measures force signals. Averaging was performed on five subjects.

**TABLE 2:** The results of different models on the test set of lower limb dataset in MEAN ± SD [minimum, maximum] (50% hold-out validation).

Model^a^	(Elderly) correlation (×100%)	(Elderly) $R^{2}\;($ ×100%)	(Elderly) adj. *R* ^2^ (×100%)	(Young) correlation (×100%)	(Young) $R^{2}\;($ ×100%)	(Young) adj. *R* ^2^ (×100%)
1	70.64 ± 13.06 [52.98, 97.29]	51.48 ± 18.95 [28.07, 94.66]	51.47 ± 18.93 [28.05, 94.65]	66.678 ± 15.91 [30.242, 89.37]	46.86 ± 20.36 [9.14, 79.95]	46.85 ± 20.34 [9.14, 79.94]
2	90.55 ± 2.11 [85.92, 92.88]	85.34 ± 4.00 [80.49, 90.80]	85.33 ± 3.97 [80.48, 90.79]	86.75 ± 15.22 [51.54, 97.93]	77.34 ± 23.20 [26.57, 95.90]	77.33 ± 23.18 [26.56, 95.89]
4	50.56 ± 31.05 [27.69, 87.98]	35.11 ± 37.17 [7.67, 77.41]	35.09 ± 37.16 [7.66, 77.40]	63.08 ± 12.46 [53.08, 77.05]	40.83 ± 16.40 [28.17, 59.36]	40.82 ± 16.38 [28.15, 59.35]
5	79.08 ± 8.39 [67.65, 95.53]	63.19 ± 13.60 [45.77, 91.25]	63.17 ± 13.58 [45.75, 91.24]	77.60 ± 11.76 [42.39, 92.19]	61.53 ± 16.44 [17.97, 85.01]	61.52 ± 16.43 [17.94, 84.99]
6	68.41 ± 0.11.63 [44.52, 86.49]	48.06 ± 15.37 [19.82, 74.81]	48.04 ± 15.35 [19.80, 74.76]	71.27 ± 13.93 [40.83, 96.33]	52.64 ± 19.52 [16.67, 92.80]	52.62 ± 19.51 [16.66, 92.78]
7	82.64 ± 13.63 [40.68, 94.28]	70.01 ± 18.34 [16.55, 88.89]	69.99 ± 18.33 [16.53, 88.86]	84.30 ± 11.23 [51.07, 93.18]	72.26 ± 16.36 [26.08, 86.82]	72.25 ± 16.34 [26.05, 86.80]
8	83.75 ± 9.31 [65.42, 94.61]	70.95 ± 14.88 [42.80, 89.52]	70.94 ± 14.86 [42.78, 89.50]	83.39 ± 9.34 [65.44, 96.40]	70.38 ± 14.97 [42.83, 92.93]	70.36 ± 14.96 [42.82, 92.91]
9	76.89 ± 14.98 [35.083, 91.16]	61.21 ± 19.91 [12.30, 83.10]	61.19 ± 19.89 [12.28, 83.08]	79.11 ± 12.42 [50.02, 98.19]	64.05 ± 18.64 [25.02, 96.41]	64.03 ± 18.62 [25.01, 96.39]
OLS	87.66 ± 10.23 [64.96, 97.35]	77.83 ± 16.81 [42.20, 94.77]	77.81 ± 16.78 [42.18, 94.75]	83.92 ± 15.922 [47.37, 98.13]	72.84 ± 24.76 [22.44, 96.31]	72.82 ± 24.75 [22.41, 96.30]
RLS	81.63 ± 16.96 [47.96, 96.63]	69.30 ± 24.79 [23.00, 93.37]	69.29 ± 24.75 [22.98, 93.35]	87.73 ± 9.55 [67.22, 97.55]	77.83 ± 16.14 [45.18, 95.16]	77.82 ± 16.11 [45.15, 95.15]
ANN	71.30 ± 23.39 [18.37, 94.85]	55.93 ± 27.72 [3.37, 89.98]	55.86 ± 27.60 [3.21, 89.96]	90.89 ± 10.14 [52.17, 98.41]	83.59 ± 15.38 [27.22, 96.86]	83.56 ± 15.24 [27.10, 96.85]
SVM	95.57 ± 2.06 [91.83, 98.22]	91.38 ± 3.93 [84.34, 96.48]	91.37 ± 3.92 [84.33, 96.47]	95.85 ± 2.29 [90.60, 98.41]	91.93 ± 4.35 [82.09, 96.86]	91.92 ± 4.34 [82.08, 96.85]
The proposed algorithm	95.64 ± 2.03 [89.525, 97.37]	91.51 ± 3.81 [80.14, 94.81]	91.50 ± 3.78 [80.13, 94.80]	97.43 ± 1.27 [94.54, 98.98]	94.95 ± 2.46 [89.39, 97.97]	94.94 ± 2.45 [89.38, 97.96]

^a^ The third model had very large values in the lower limb dataset that resulted in zero goodness-of-fit in all indices. The Correlation Coefficient and Goodness-of-fit measures (*R* ^2^ and adj. *R* ^2^) were calculated between the reconstructed and measured force signals. Averaging was performed on 19 and 14 subjects in the young and elderly groups.

**FIGURE 2:**
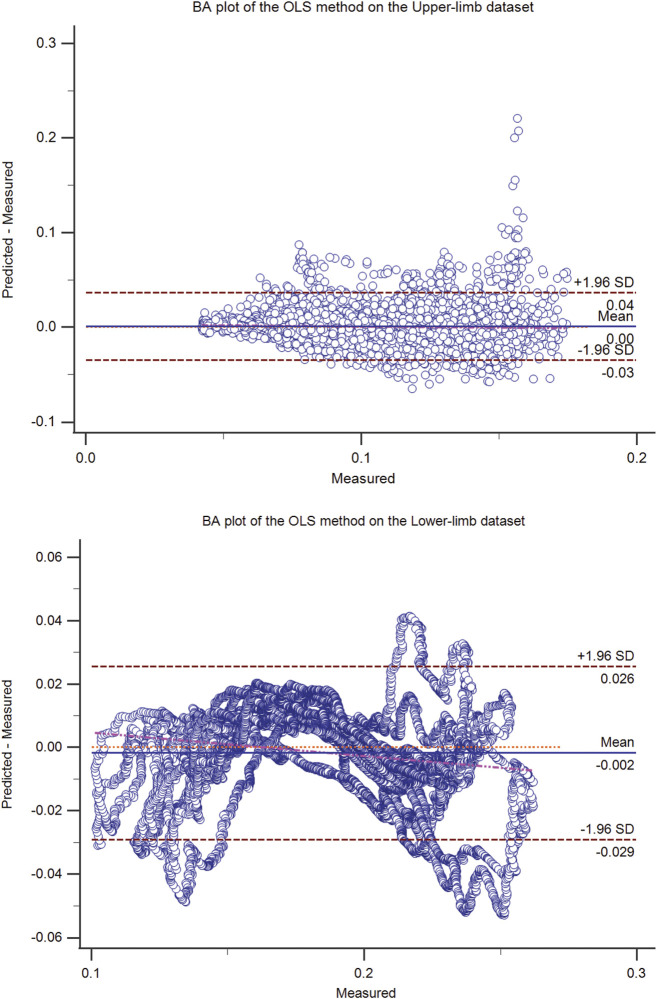
The Bland-Altman (BA) plot of the OLS method on the upper-limb (top) and the lower-limb (bottom) test dataset (50% hold-out validation). The BA plot shows the scatter plot of the residual signal (predicted force minus measured force signal) (y-axis) and the measured force signal (x-axis), which identifies the homogeneity of the residual signal at different measured signal levels. In addition to the mean residual signal (i.e., bias), the upper and lower boundaries (mean (residual signal) ± SD (residual signal)) are also shown.

**FIGURE 3:**
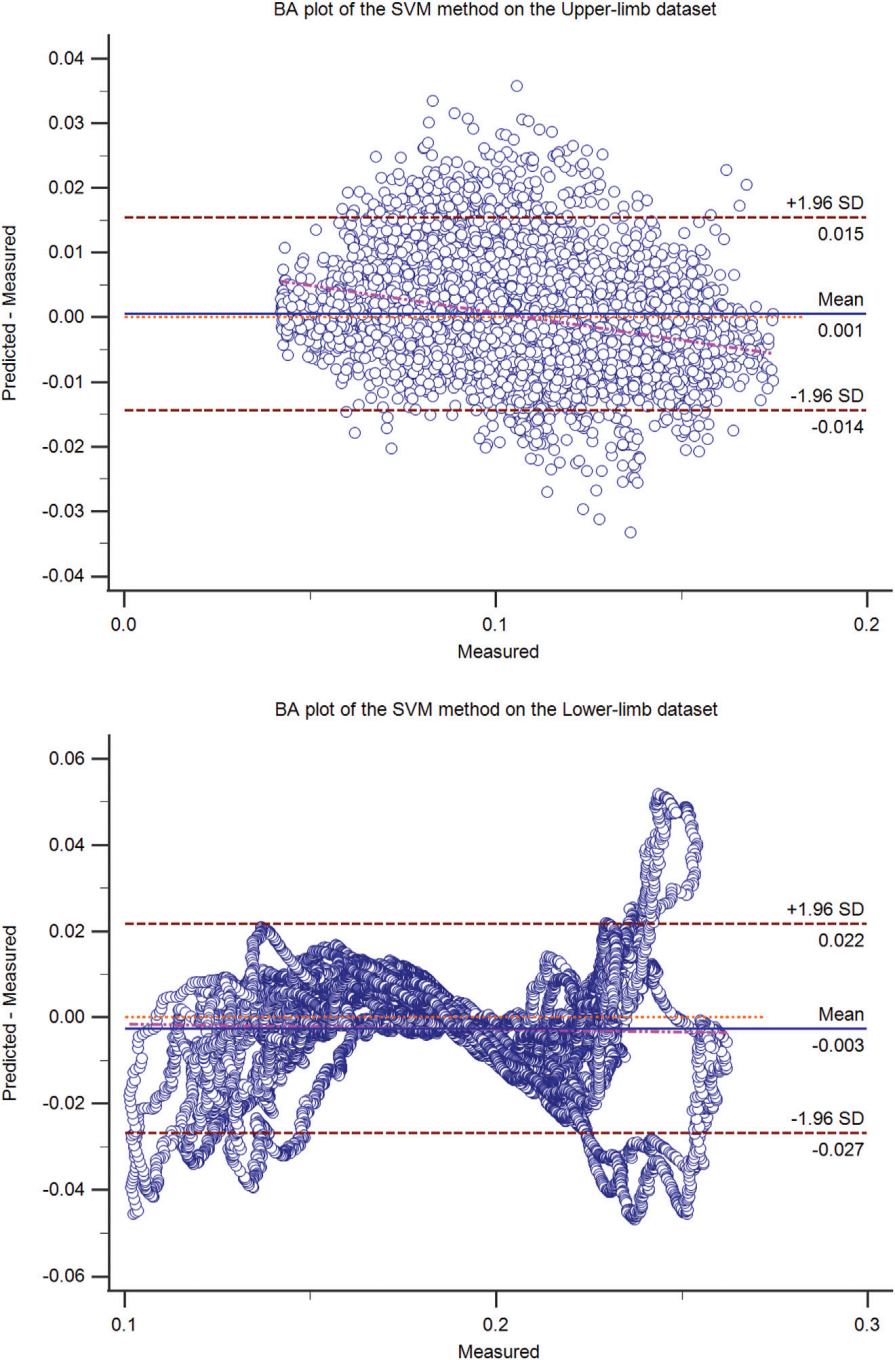
The Bland-Altman (BA) plot of the SVM method on the upper-limb (top) and the lower-limb (bottom) test dataset (50% hold-out validation). The BA plot shows the scatter plot of the residual signal (predicted force minus measured force signal) (y-axis) and the measured force signal (x-axis), which identifies the homogeneity of the residual signal at different measured signal levels. In addition to the mean residual signal (i.e., bias), the upper and lower boundaries (mean (residual signal) ± SD (residual signal)) are also shown.

**FIGURE 4:**
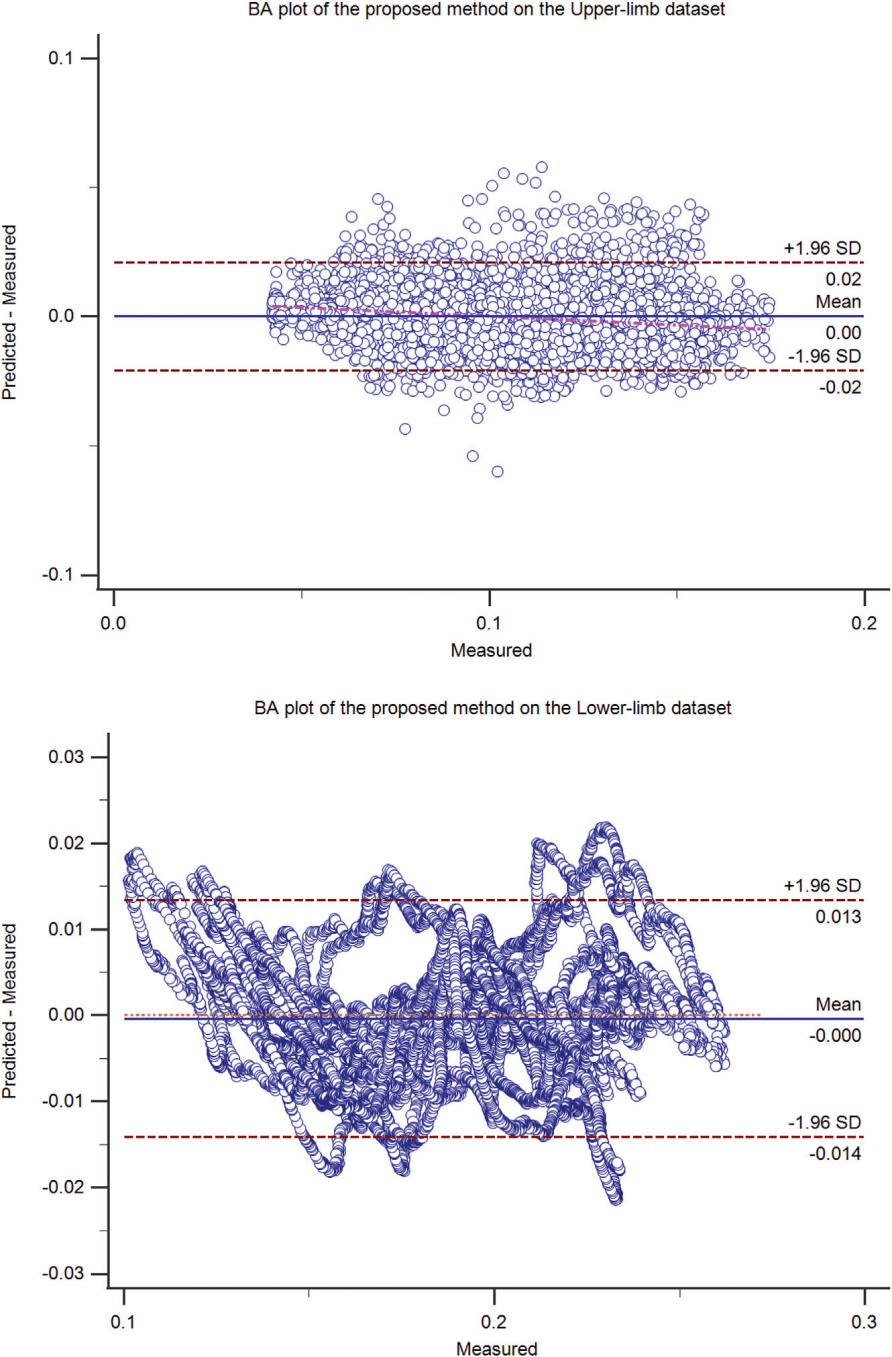
The Bland-Altman (BA) plot of the proposed method on the upper-limb (top) and the lower-limb (bottom) test dataset (50% hold-out validation). The BA plot shows the scatter plot of the residual signal (predicted force minus measured force signal) (y-axis) and the measured force signal (x-axis), which identifies the homogeneity of the residual signal at different measured signal levels. In addition to the mean residual signal (i.e., bias), the upper and lower boundaries (mean (residual signal) ± SD (residual signal)) are also shown.

The residual signal was further analyzed using the Bland-Altman plot. The Bland Altman plot of the best models (ANN, OLS, SVM, and the proposed method) was shown in Figures 1–4 for upper and lower limbs datasets. Since the OLS and RLS methods had comparable results, The Bland Altman plot of the OLS method was provided.

**FIGURE 5:**
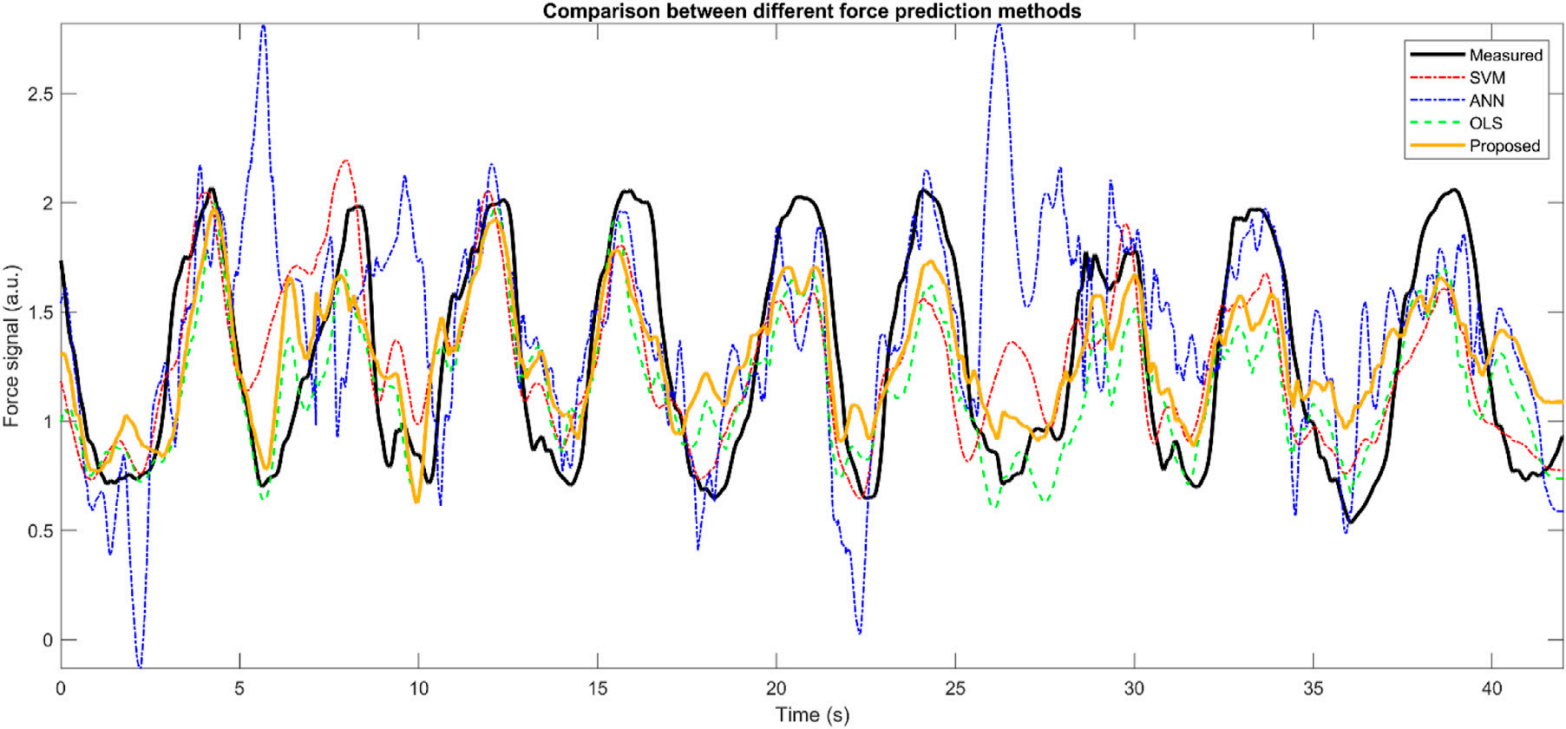
The comparison between ANN, OLS, SVM and the proposed method on a sample data from the lower-limb test dataset (50% hold-out validation).

The goodness-of-fit of ANN, OLS, SVM, and the proposed method is shown on a sample recording from the upper-limb dataset (Figure 5).

**FIGURE 6:**
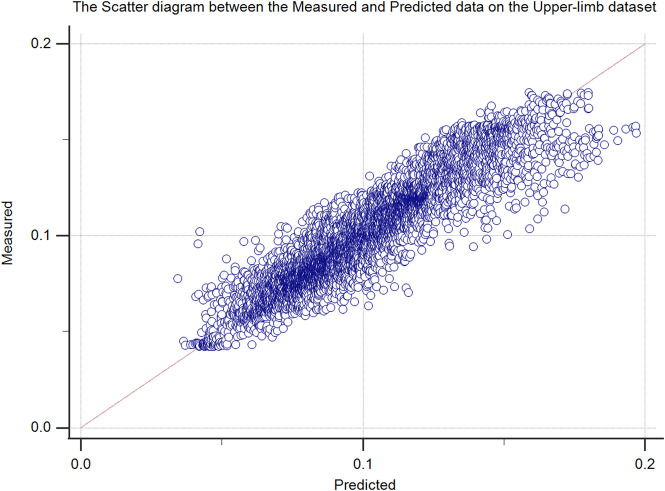
The scatter plot of the proposed method (measured vs. predicted force data) upper-limb test dataset (50% hold-out validation).

**FIGURE 7:**
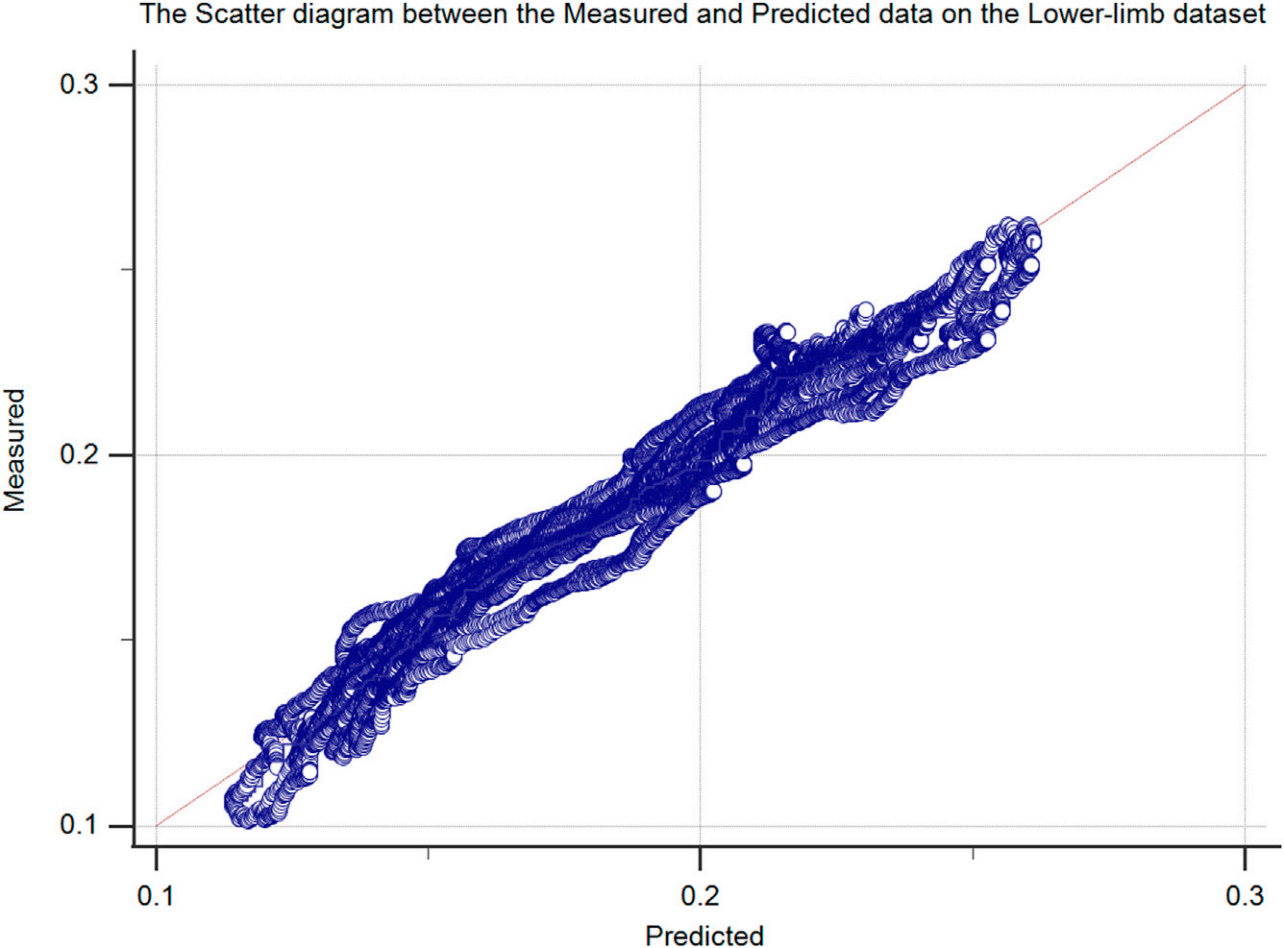
The scatter plot of the proposed method (measured vs. predicted force data) lower-limb test dataset (50% hold-out validation).

**FIGURE 8:**
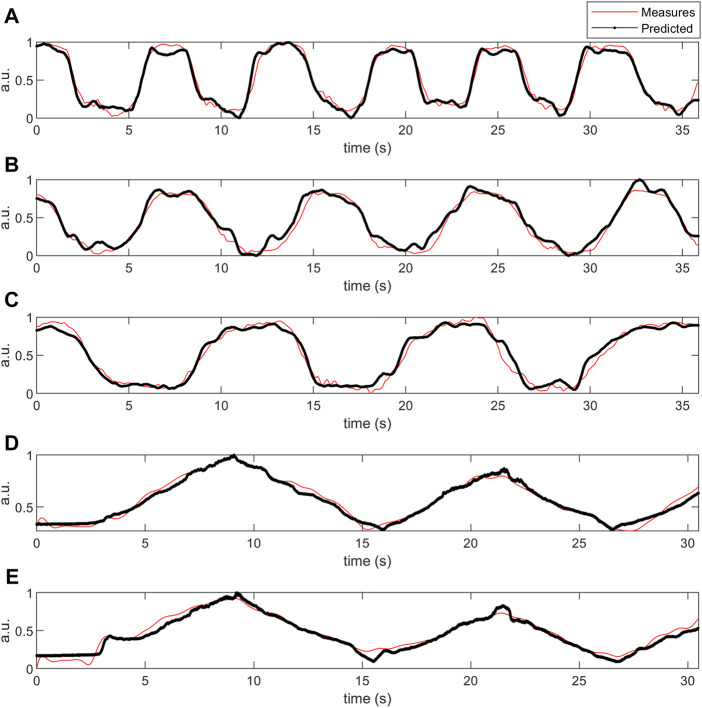
The measured and predicted force signals in different subjects from upper **(D, E)** and lower limb **(A–C)** test datasets (50% hold-out validation). The y-label data was normalized and had normalized arbitrary units (a.u.).

The proposed method was further analyzed regarding the scatter plot between the predicted and measured force data in the upper (Figure 6) and lower-limb (Figure 7) datasets. The predicted signals of the proposed method and the measured force signals in different subjects from the upper and lower limb datasets are provided in Figure 8.

**FIGURE 9:**
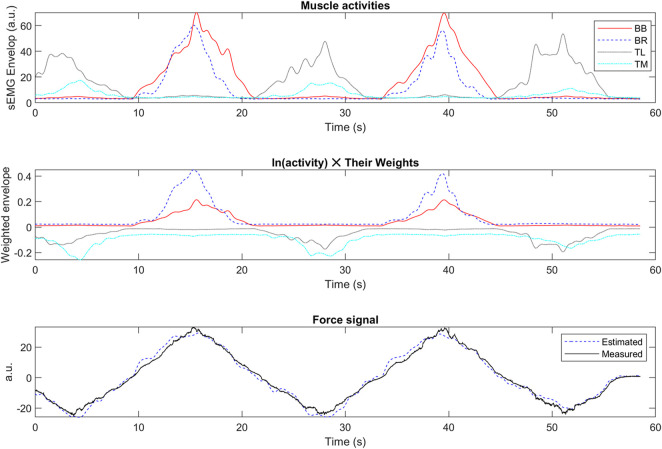
The envelope of the sEMG signal (top), the weighted activity of each muscle estimated from Eq. 7 (middle), and the estimated v.s. measured force signals (bottom) in sample data from the upper-limb test dataset (50% hold-out validation). The y-label data has a normalized arbitrary unit (a.u.).

**FIGURE 10:**
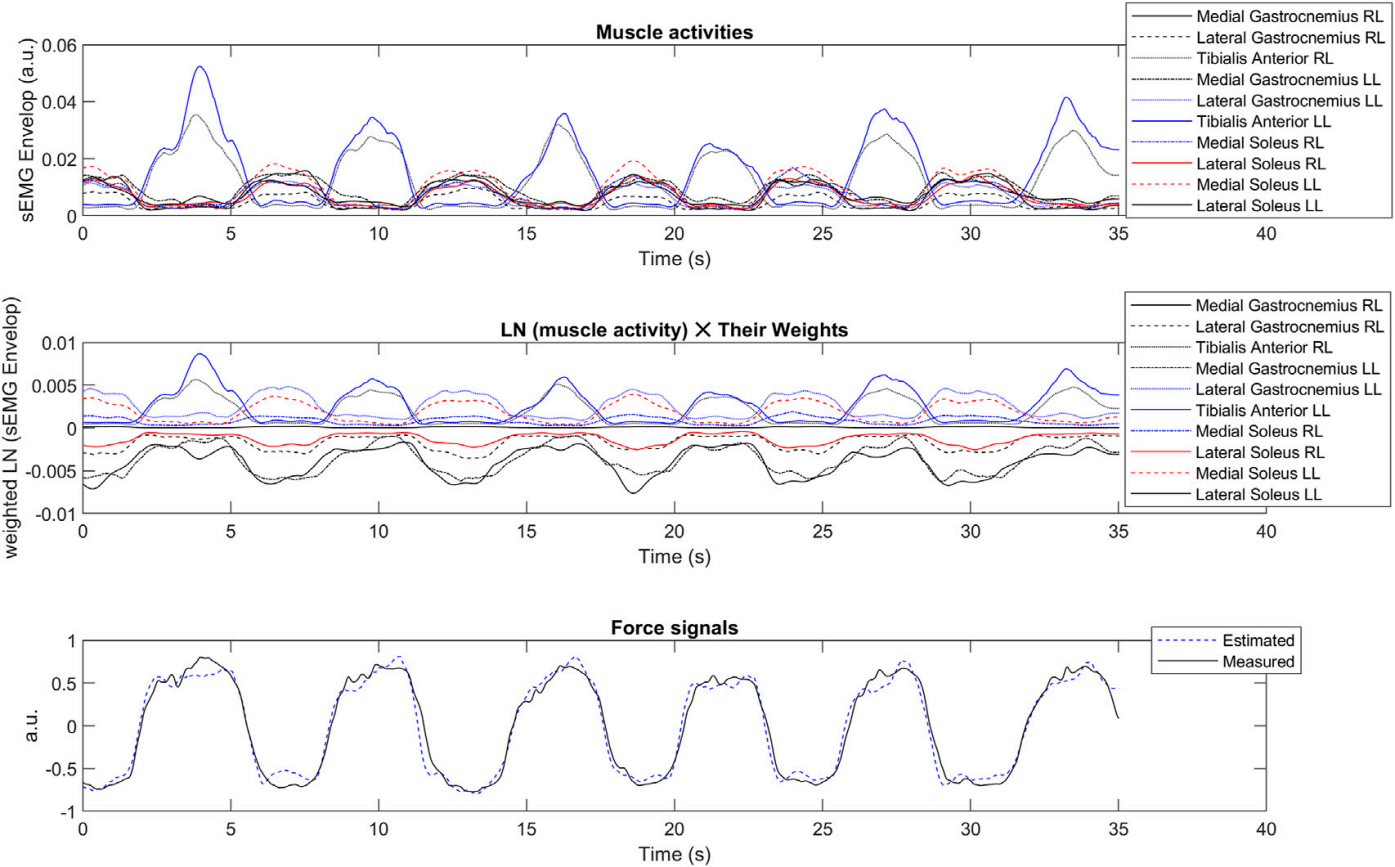
The envelope of the sEMG signal (top), the weighted activity of each muscle estimated from Eq. 7 (middle), and the estimated v.s. measured force signals (bottom) in a sample data from the lower-limb test dataset (50% hold-out validation). The y-label data has normalized arbitrary unit (a.u.). RL: Right Leg; LL: Left Leg; LN: Natural Logarithm.

The envelope of the sEMG signal, the weighted activity of each muscle estimated from Eq. 7, and the estimated vs. measured force signals were provided for the upper (Figure 9) and lower-limb (Figure 10) datasets.

The average running time of each 250 msec-epoch signal of the training and testing of the proposed method was 30.3 ± 3.4 [27.6, 35.9] and 6.7 ± 2.2 [4.7, 9.5] for the upper limb and 25.1 ± 3.6 [22.3, 40.8] and 11.6 ± 2.4 [8.5, 17.8] for the lower-limb dataset in us.

The weights of the muscle’s tibialis anterior (right leg), medical soleus (left leg), and the intercept point were statistically significant in the minimally active and active groups (*p*-value < 0.05). The ROC curve of these three indicators was provided in Supplementary Figure S1. Their AUC was 0.722, 0.574, and 0.914. The intercept point significantly outperformed the medical soleus (left leg) in terms of AUC (*p*-value < 0.05), while the intercept point and the tibialis anterior (right leg) were comparable. The proposed model could discriminate between active and minimally active subjects of the lower limb dataset with Type I and II errors of 0.22 and 0.00, respectively.

## 5 Discussion

### 5.1 The performance of the proposed method

The proposed method had the minimum *adj. R* ^2^ of 80.13% in the entire test sets of the upper and lower-limb datasets (Tables 1, 2), showing a proper goodness-of-fit (Figures 4–10). Since the number of samples was much more than the number of parameters of the models, *adj. R* ^2^ and *R* ^2^ indices were very similar (Eq. 22; Tables 1, 2). Based on the Bland Altman plots of Figure 4), the residual signal of the proposed method has better homogenous variance in the upper-limb dataset compared with the lower-limb dataset. The regression line of the residual signal of the upper-limb dataset was y = 0.006848–0.06733×x, while it was y = −0.0003940 + 0.00007351×x for the lower-limb dataset, where parameters *x* and *y* are the measured and residual force signals.

The regression line of the scatter plots of Figures 6, 7 were y = 0.008371 + 0.9175 ×x (*R* ^2^ = 85.6%), and y = 0.007631 + 0.9612×x (*R* ^2^ = 96.1%), respectively, showing proper fitness of the predicted force signal (x) vs. the measured signal (y) in the upper and lower-limb datasets. In the entire datasets, the maximum running time of the proposed algorithm was 40.8 us and 17.5 us on the training and testing datasets, respectively. Thus, it is suitable for real-time applications.

The cut-off frequency of 2.0 Hz was used in our study for envelope estimation. The sEMG amplitude during isometric and quasi-static contractions was shown to have a frequency below 5–10 Hz, and the optimal cut-off frequencies between 2–3 Hz were provided in the literature (Staudenmann et al., 2010; Ranaldi et al., 2022). Such a cut-off frequency compromises sEMG dynamics, muscle force difference, and the time lag between those signals (Staudenmann et al., 2010). Different cut-off frequencies could affect the smoothness resulting in correlations among estimated sEMG amplitude and any target signal, especially in non-slowly varying signals (Ranaldi et al., 2022). Moreover, non-causal digital filters were used in our study. In a real-time application, however, causal filters must be used. Since both filters of the sEMG and muscle force were non-causal, such a modification should not be problematic.

Squared or absolute sEMG signals could be used for envelope detection. While the former is optimal for normal distribution, the latter is used when the distribution of the sEMG signals is more centrally peaked (a.k.a., Laplacian) (Hogan and Mann, 1980; Clancy and Hogan, 1999; Clancy et al., 2004). The distribution of our signal epochs was mostly Laplacian, as identified by Goodness-of-fit Test Statistics for the Laplace Distribution (Puig and Stephens, 2000). Moreover, the variation of the absolute signal is lower than that of the squared signal, resulting in a superior signal-to-noise ratio (Clancy and Hogan, 1999). Thus, the absolute signal (a.k.a., full-wave rectifier) was used in our study for envelope detection.

The proposed algorithm is a convex representation of models 2 and 4, in which the natural logarithm of the sEMG envelopes is used. The logarithmic transformation is primarily used in statistics to reduce the skewness of the data (West, 2022), resulting in a more normally distributed dataset, which is preferred in clinical practice (Feng et al., 2014). Such a transformation is also helpful to use LS, a convex optimization method, rather than stochastic optimization methods (e.g., PSO), which are time-consuming and get stuck in local minima (Beck, 2014) (Tables 1, 2). Moreover, the logarithmic transformation, a member of the Box-Cox family of transformations, could improve the performance of the regression models in system identification (Keene, 1995; Ljung, 1999).

The proposed model could classify active and minimally active subjects of the lower limb dataset, mainly based on the importance of the tibialis anterior muscle during sway protocol. The algorithm’s sensitivity is 100% to identify active subjects, while its specificity is 78% to detect minimally-active subjects. Tibialis anterior muscle was shown as an essential muscle during quiet standing sway in healthy and also subjects with neurodegenerative diseases (such as Parkinson’s disease) (Vette et al., 2017) (Warnica et al., 2014). The soleus is one of the principal sources of proprioceptive information during standing (Di Giulio et al., 2009), whose exercises might help to reduce the risk of falling in the elderly (Lee and Yoo, 2017). Figure 10 shows that the tibialis anterior (RL) or medial soleus (LL) are mainly active during sway protocol. They are the antagonist’s muscles and have important roles in postural control (Nagai et al., 2012).

### 5.2 The stable solution to the LS problem

In the proposed algorithm, the matrix A in Eq. 6 is estimated using the envelope of the sEMG signals (Eq. 8). We used the stable solution of Eq. 6 (i.e., A\b) instead of calculating the inverse matrix (A^T^A)^−1,^ which is highly affected by the condition number of the matrix (Pintelon and Schoukens, 2012; Beck, 2014). The condition number of the matrix A equals the square root of the condition number of the matrix (A^T^A). The condition number of the positive semidefinite matrix (A^T^A) equals the maximum eigenvalue of (A^T^A) divided by the minimum eigenvalue of (A^T^A) (Beck, 2014).

We further calculated the correlation between the *adj. R* ^2^ and the condition number of the matrix A (Eqs 6, 8). The average condition number of matrix A was 763 ± 464. No significant correlation was found between the goodness-of-fit and the condition number of the matrix in the entire dataset (*r* = −0.263; *p*-value = 110). Our method not only had proper goodness-of-fit but also did not have a significant bias in the entire dataset (*p*-value = 0.610).

In our model (Eq. 7), we also estimated the intercept point (w_0_). If it is subtracted from the measured force signal (f(t)), the mean value of the measured signal is removed, and the number of estimated parameters is decreased. It is aligned with the recommendation of the system identification guidelines to remove the measured signals detrend before system identification.

### 5.3 The grey-box structure

The same LS model was used in our method for the upper and lower-limb datasets, showing the generalization capability of the proposed algorithm. However, our method’s goodness-of-fit and residual signal analysis were better on the upper-limb dataset than on the lower-limbs dataset (Tables 1, 2; Figure 4). It could be that the number of involved muscles in the upper-limb dataset was less than that of the lower-limb dataset. Moreover, unlike black-box models such as ANN or SVM (Mokri et al., 2022), our method is a grey-box model, in which the model interpretation is possible (Figures 9, 10). The activity of each muscle could be provided for each active muscle during recording to provide insights into load-sharing problems (Rojas-Martínez et al., 2019). Such information is also helpful in prosthesis control to identify major active muscles when a limited number of electrodes are used in practice.

### 5.4 Comparison with the state-of-the-art

Traditional EMG amplitude processing methods were used for comparison with our proposed model. Since analyzed methods had statistically significantly different results using the rm-ANOVA (F (4,567,728) = 969.856; *p*-value < 0.001), the pairwise comparison was performed, and to reduce the Type I error, the Bonferroni correction was used in our study and *adj. p*-value was reported. Overall, the proposed method significantly outperformed the other methods (*adj. p*-value < 0.05), in terms of the goodness-of-fit measures. The comparison between ANN, OLS, and SVM methods and the proposed method on a recording from the upper-limb dataset was further provided in Figure 5. ANN does not provide proper fitness among the analyzed models, as shown in the Bland-Altman plot (Figure 1). It could be because the ANN method is a black-box method, and they are not usually acceptable in clinical applications.

The OLS did not have a good Bland Altman plot either (Figure 2), showing that linear weights were unsuitable for the EMG-force problem. However, OLS (a.k.a. Multiple Linear Regression) has been widely used in the literature. It was shown in the literature that the EMG-force relationship (i.e., the sEMG envelope, which is smoothed rectified EMG by itself, and muscle force) needs not be linear (Fuglevand et al., 1993; Hof, 1997), especially in broad force range. The muscle force has different biomechanical components, including the sigmoid shape between excitation and muscle force (Fuglevand et al., 1993), which could be approximated using the exponential forms.

### 5.5 Limitations and future activities

One of the limitations of our study is that we did not use a random force trajectory. It was shown that such a force profile could provide rich information about muscle excitation (Wang et al., 2021), which is the focus of our future work. Using proper signal processing methods, it is possible to identify not only the frequency spectrum of the trajectory force signal to stimulate different frequencies equally, but we can also provide training and test sets without a medium-to-high degree of similarity to reduce such a bias. However, the average correlation between the epochs of estimation and test sets of the measured force signals was −0.0046 ± 0.6812 and −0.0048 ± 0.5194 in the upper and lower limbs datasets, respectively, showing low-to-medium correlation of the measured force signal in estimation and test sets in our study.

Also, the analyzed methods did not consider the electromechanical delay (Lacourpaille et al., 2013). Although properly incorporating the delay parameter could improve the goodness-of-fit of the models (Johns et al., 2016), it increases the complexity of the methods (Corcos et al., 1992; Isermann and Münchhof, 2011) that might not be suitable for real-time applications. Having implemented classical signal conditioning methods on the sEMG signals, a delay is introduced as a solution to this problem, although not a systematic method. An accurate and efficient estimation of this parameter will also be a focus of our future activities. Moreover, in our study, a representative sEMG channel was selected for each muscle. Alternatively, principal component analysis (PCA) could improve the force estimation from HDsEMG signals (Staudenmann et al., 2006).

Moreover, we did not compare our method with sEMG superimposition, which uses motor control information rather than traditional EMG amplitude processing, which has been recently used in muscle force estimation (Savc et al., 2018). In principle, it is possible to use parts of the sEMG decomposition algorithms (such as, (Mohebian et al., 2019) to estimate muscle activity index as to estimate muscle force. However, the running time and the required number of electrodes of sEMG decomposition-based methods are problematic in real-time force prediction compared with fast and reliable EMG amplitude-based methods, such as our proposed method.

It was shown in the literature that amplitude cancellation distorts the spectrum of the rectified sEMG signal (Dideriksen and Farina, 2019). Moreover, many factors influence the relation between EMG amplitude and force, including the amount of crosstalk from nearby muscles, the number and the discharge rate of recruited motor units, Skin-electrode contact, Interelectrode distance, Electrode size and shape, the orientation of the recording array compared with the muscle fibers, Length of the fibers, the shape of the volume conductor, and thickness and inhomogeneities of the subcutaneous tissue layers. Moreover, the same control strategy may generate signals with different amplitude trends based on the motor unit locations (Farina et al., 2004). Moreover, optimal smoothing and envelope detection depends on the load-sharing scenario (Staudenmann et al., 2010). Thus, a specific sEMG amplitude-force relation cannot have general validity and could have a subject-by-subject and muscle-by-muscle relationship. It was also shown in the literature that the relationship between force and absolute sEMG is linear for small muscles with narrow motor unit recruitment force ranges, while it is non-linear for larger muscles with wide motor unit recruitment force ranges (such as proximal leg or arm muscles) (Zhou and Rymer, 2004).

However, our proposed method had an average goodness-of-fit (*R* ^2^) of 96.77 ± 1.67 (%) and 91.08 ± 6.84 (%) for the upper and lower-limb datasets, respectively. The subjects-by-subject variations are acceptable, as SD values are less than the uncertainty on the mean values (i.e., MEAN divided by the number of subjects).

The primary elbow flexors are the biceps brachii, brachialis, and brachioradialis, while the triceps brachii is the primary extensor. Moreover, anconeus could contribute to elbow extension (Day, 2009). The brachialis muscle is a fusiform muscle located deep to the biceps brachii. Since the sEMG of such deep muscles cannot be collected with surface electrodes (e.g., brachialis muscle) remains a substantial limitation and a significant cause of the error (Botter et al., 2011). However, it could be assumed that the approximation error term includes the activity of the deep muscle if such an activity is not correlated with other agonist or antagonist muscles since the residuals must be uncorrelated with the predictors in regression analysis. Triceps brachii is the antagonist, and brachialis is a synergist with biceps brachii. Thus, the condition applies to elbow flexion and extension.

For the lower limb muscles, we did not miss any major muscles concurring with the production of plantar flexion torque. Moreover, during quiet standing, dorsiflexors are well-acknowledged to be silent (Di Giulio et al., 2009). Therefore, the sEMG signals we collected from gastrocnemii and soleus represent the net excitation commanding plantar flexion. However, our sEMG signals were likely not sensitive to changes in excitation of the proximal soleus region, which is covered by the gastrocnemius heads. Of more critical concern, though, is collecting sEMG sensitive to mediolateral differences in soleus excitation, which have been shown to change during quiet and perturbed standing (Cohen et al., 2020; Cohen et al., 2021). With our experimental protocol, sEMG signals were most sensitive and specific to the target muscles, minimizing both Types I and II errors (Vieira and Botter, 2021).

The required sample size of the experiment was calculated based on the expected goodness-of-fit (e.g., R2), the number of predictors, and the statistical power in our regression analysis (Alasuutari et al., 2008; Stanley, 2022). For the upper limb dataset, with the expected *R* ^2^ of 0.964 (Jafari et al., 2014), five variables, and a statistical power of 80%, the minimum sample size was 8. For the lower limb dataset, however, with the expected *R* ^2^ of 0.960 (Mokri et al., 2022), 11 variables, and a statistical power of 80%, the minimum sample size was 14. In our study, five subjects participated in the upper limb study, while 33 subjects were enrolled in the lower limb study. Thus, the reliability of generalization of the lower-limb experiment is expected, while this is not fully guaranteed in the upper-limb dataset.

## 6 Conclusion

In conclusion, we proposed a real-time grey-box model to estimate muscle forces recorded at the joints. The goodness-of-fit, residual signal analysis, and rigorous statistical comparison with the state-of-the-art on the upper-limb and lower-limb datasets showed that the proposed method is promising.

## Data Availability

The original contributions presented in the study are included in the article/Supplementary Materials, further inquiries can be directed to the corresponding author.
