# Supplementary material for: A real-time and convex model for the estimation of muscle force from surface electromyographic signals in the upper and lower limbs
Source: Front Physiol. 2023 Feb 27;14:1098225. doi: 10.3389/fphys.2023.1098225 (PMC10009160; doi:10.3389/fphys.2023.1098225)
Supplement: Supplementary file 1 [file Table1.docx]

Table s1: The literature review of the performance indices used in recent EMG-force models.

| **Authors** | **Muscle** | **#Subjects** | **Model** | **Validation Framework** | **Performance Indices** | **Regression Diagnostics** | **Statistical Method** |
| --- | --- | --- | --- | --- | --- | --- | --- |
| (Su et al., 2021) | forearm | 1 | NN (Deep Learning) | Hold out | MAE | - | - |
| (Ma et al., 2020) | Forearm | 3 Healthy | Gene Expression Programming | - | RMSE, CC | RMSE diagram and plot | - |
| (Chen et al., 2020) | forearm | 7 Healthy | NN | 1 DOF for training and 3DOF for test | RMSE | RMSE diagram | RANOVA and for post hoc they used paired t-test with Bonferroni correction |
| (Rane et al., 2019) | Lower limb muscles | 156 | NN | Hold out (80% training, 10% validation, 10% test) | RMSE | RMSE Plot | - |
| (Xu et al., 2018) | biceps brachii | 8 Healthy | NN | Hold out (80% training, 10% validation, 10% test) | RMSE | Bland-Altman plots, RMSE boxplot | Kruskal-Wallis one-way analysis of variance |
| (Huang et al., 2017) | biceps brachii | 12 Healthy | nonnegative matrix factorization | Hold out (one repetition from five repetitions as training and others for test) | RMSD, VAF | Bland–Altman plots,  RMSD plots | RANOVA |
| (Cao et al., 2017) | forearm | 10 Healthy | extreme learning machine, SVM, multiple nonlinear regression | Hold out (90% training, 10% test) | RMSE and CC | RMSE plot | - |
| (Na and Kim, 2016) | Bíceps, brachioradialis | 10 Healthy | A new proposed model based on muscle-twitch model | Hold out | RMSE, $\boldsymbol{R}^{\boldsymbol{2}}$ | RMSE and $\boldsymbol{R}^{\boldsymbol{2}}$ plots | two-sample t-test |
| (Hashemi et al., 2014) | biceps brachii and triceps brachii | 10 Healthy | Parallel cascade identification | Hold out | RMSE | RMSE Plot | Wilcoxon signed-rank test |
| (Li et al., 2014) | agonist and antagonist muscles | 5 Healthy | Demodulation | Two-fold CV | - | RMS of error | - |
| (Hashemi et al., 2013) | biceps brachii | 9 Healthy | Hill muscle model | Hold out | RMSE | RMSE boxplot | one-way ANOVA |
| (Hayashibe and Guiraud, 2013) | biceps brachii, brachioradialis and triceps brachii | 10 Healthy | parallel cascade identification | Hold out | RMSE | RMSE boxplot | - |

MAE: Mean Absolute Error; RMSE: Root Mean Square Error; CC: Correlation Coefficient; RMSD: Root mean square difference; VAF: Variance Accounted For; ANOVA: Analysis of variance; CV: Cross Validation; NN: Neural Network;


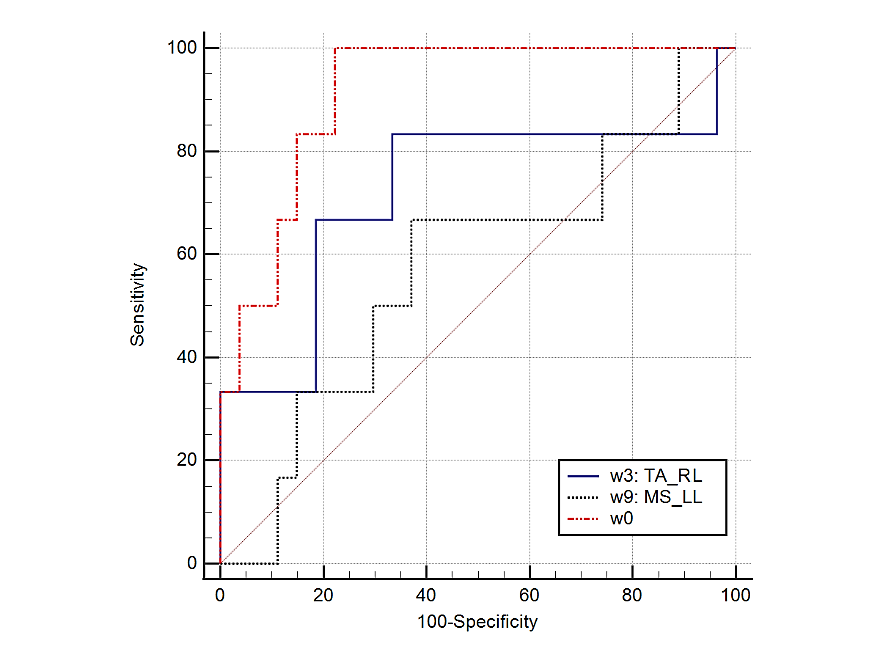


Figure s1: The comparison between the Receiver operating characteristic (ROC) curve of the weights of the muscle’s tibialis anterior (right leg)(TA_RL), medical soleus (left leg)(MS_LL), and the intercept point (w0) to classify minimally active and active groups in the lower limb dataset.

References

CAO, H., SUN, S. & ZHANG, K. 2017. Modified EMG-based handgrip force prediction using extreme learning machine. *Soft computing,* 21**,** 491-500.

CHEN, Y., DAI, C. & CHEN, W. 2020. Cross-Comparison of EMG-to-Force Methods for Multi-DoF Finger Force Prediction Using One-DoF Training. *IEEE Access,* 8**,** 13958-13968.

HASHEMI, J., MORIN, E., MOUSAVI, P. & HASHTRUDI-ZAAD, K. 2013. Surface EMG force modeling with joint angle based calibration. *Journal of Electromyography and Kinesiology,* 23**,** 416-424.

HASHEMI, J., MORIN, E., MOUSAVI, P. & HASHTRUDI-ZAAD, K. 2014. Enhanced dynamic EMG-force estimation through calibration and PCI modeling. *IEEE Transactions on Neural Systems and Rehabilitation Engineering,* 23**,** 41-50.

HAYASHIBE, M. & GUIRAUD, D. 2013. Voluntary EMG-to-force estimation with a multi-scale physiological muscle model. *BioMedical Engineering OnLine,* 12**,** 86.

HUANG, C., CHEN, X., CAO, S., QIU, B. & ZHANG, X. 2017. An isometric muscle force estimation framework based on a high-density surface EMG array and an NMF algorithm. *Journal of neural engineering,* 14**,** 046005.

LI, Z., WANG, B., SUN, F., YANG, C., XIE, Q. & ZHANG, W. 2014. sEMG-Based Joint Force Control for an Upper-Limb Power-Assist Exoskeleton Robot. *IEEE Journal of Biomedical and Health Informatics,* 18**,** 1043-1050.

MA, R., ZHANG, L., LI, G., JIANG, D., XU, S. & CHEN, D. 2020. Grasping force prediction based on sEMG signals. *Alexandria Engineering Journal*.

NA, Y. & KIM, J. 2016. Dynamic elbow flexion force estimation through a muscle twitch model and sEMG in a fatigue condition. *IEEE Transactions on Neural Systems and Rehabilitation Engineering,* 25**,** 1431-1439.

RANE, L., DING, Z., MCGREGOR, A. H. & BULL, A. M. 2019. Deep learning for musculoskeletal force prediction. *Annals of biomedical engineering,* 47**,** 778-789.

SU, H., QI, W., LI, Z., CHEN, Z., FERRIGNO, G. & MOMI, E. D. 2021. Deep Neural Network Approach in EMG-Based Force Estimation for Human–Robot Interaction. *IEEE Transactions on Artificial Intelligence,* 2**,** 404-412.

XU, L., CHEN, X., CAO, S., ZHANG, X. & CHEN, X. 2018. Feasibility study of advanced neural networks applied to sEMG-based force estimation. *Sensors,* 18**,** 3226.
